# Outer Surface Protein B Is Critical for Borrelia burgdorferi Adherence and Survival within *Ixodes* Ticks

**DOI:** 10.1371/journal.ppat.0030033

**Published:** 2007-03-09

**Authors:** Girish Neelakanta, Xin Li, Utpal Pal, Xianzhong Liu, Deborah S Beck, Kathleen DePonte, Durland Fish, Fred S Kantor, Erol Fikrig

**Affiliations:** 1 Section of Rheumatology, Yale University School of Medicine, New Haven, Connecticut, United States of America; 2 Section of Allergy and Immunology, Department of Internal Medicine, Yale University School of Medicine, New Haven, Connecticut, United States of America; 3 Department of Epidemiology and Public Health, Yale University School of Medicine, New Haven, Connecticut, United States of America; University of Chicago, United States of America

## Abstract

Survival of Borrelia burgdorferi in ticks and mammals is facilitated, at least in part, by the selective expression of lipoproteins. Outer surface protein (Osp) A participates in spirochete adherence to the tick gut. As *ospB* is expressed on a bicistronic operon with *ospA,* we have now investigated the role of OspB by generating an OspB-deficient B. burgdorferi and examining its phenotype throughout the spirochete life cycle. Similar to wild-type isolates, the OspB-deficient B. burgdorferi were able to readily infect and persist in mice. OspB-deficient B. burgdorferi were capable of migrating to the feeding ticks but had an impaired ability to adhere to the tick gut and survive within the vector. Furthermore, the OspB-deficient B. burgdorferi bound poorly to tick gut extracts. The complementation of the OspB-deficient spirochete in trans, with a wild-type copy of *ospB* gene, restored its ability to bind tick gut. Taken together, these data suggest that OspB has an important role within Ixodes scapularis and that B. burgdorferi relies upon multiple genes to efficiently persist in ticks.

## Author Summary

Lyme disease is the most common vector-borne disease in North America and Europe. The causative agent Borrelia burgdorferi is a bacterium that is maintained in an enzoonotic cycle between *Ixodes* ticks and a large range of mammals. Accidental encounters of infected *Ixodes* ticks with humans results in the transmission of B. burgdorferi and subsequent Lyme disease. Given that global control efforts have met with limited success, the need for developing novel interventions to combat this infection has become all the more vital. A better understanding of how B. burgdorferi interacts with its vector might lead to new ideas for combating the Lyme disease. B. burgdorferi upregulates outer surface protein (Osp) A and B during entry into ticks, and OspA contributes to the colonization of bacterium within the vector gut. We now demonstrate that OspB also facilitates the colonization and survival of B. burgdorferi in ticks. This work provides the basis for future studies as to how this protein facilitates interaction of B. burgdorferi to the tick gut and thus ultimately a basis for the development of novel strategies to interrupt the spirochete life cycle.

## Introduction

Lyme disease is the most common tick-borne disease in the United States [1]*.* The causative organism, *Borrelia burgdorferi,* is a microaerophilic spirochete that contains a 910-kb linear chromosome and at least 21 linear and circular plasmids [2]. B. burgdorferi is composed of several genospecies known collectively as B. burgdorferi sensu lato (s.l.) of which B. burgdorferi sensu stricto, *Borrelia garinii,* and Borrelia afzelii are responsible for most cases of Lyme borrelliosis worldwide [3,4]. B. burgdorferi s.l. is maintained in an enzootic cycle that primarily involves *Ixodes* ticks and a large range of transmission-competent vertebrate hosts [1, 3–6]. Ticks of the Ixodes ricinus species complex, including Ixodes scapularis and Ixodes pacificus in eastern and western North America, respectively [3], and I. ricinus and Ixodes persulcatus in Europe and Eurasia [4], respectively, are competent vectors for the transmission of B. burgdorferi s.l. during engorgement on a reservoir host [1,3–6]. Studies reported so far tend to show similar means of transmission and modes of pathogenesis of the B. burgdorferi s.l. in this group of ticks [3–8]. After entry into the ticks, B. burgdorferi s.l. replicates and persists within the gut, then during a subsequent blood meal, migrates through the vector and is transmitted to a new host [9]. In humans, B. burgdorferi s.l. initially establishes a localized infection in the skin at the site of the tick bite known as *erythema migrans,* then disseminates via the blood stream and can chronically infect distant organs, resulting in arthritis, carditis, and neurological disease [1,10]. Laboratory mice can be infected with B. burgdorferi s.l. and serve as a reliable model for the study of Lyme borreliosis [11].

Variation in the synthesis of outer surface proteins (Osps) is a primary strategy by which B. burgdorferi evades the host immune system and adapts to various host microenvironments, such as those in a mammal or a tick vector [12–15]. Numerous studies have shown that B. burgdorferi selectively expresses specific Osps in distinct phases of its life cycle and in specific tissue locations. For example, the expression of B. burgdorferi OspA and OspB is immediately turned on when the spirochetes enter and reside within the arthropod vector. However, during transmission from the arthropod vector to a vertebrate host, B. burgdorferi downregulate OspA and OspB expression and upregulate the expression of proteins such as OspC, DbpA, and BBK32 [16–21]. This selective and temporal gene expression of OspA and OspB in ticks suggests that these two proteins may function during early spirochete colonization and persistence within the tick vector. Indeed, a recent study showing that OspA mediates spirochete adherence within the tick gut by binding to the I. scapularis TROSPA protein [22] supports this contention and indicates how stage-specific gene expression contributes to the maintenance of the natural cycle of the spirochete.

The genes *ospA* and *ospB* are highly conserved among B. burgdorferi isolates in the United States [23,24]. They are encoded on the linear plasmid (lp) 54 and are generally expressed by a common promoter [25,26]. Both OspA and OspB are surface-exposed lipoproteins that are closely related in terms of sequence and structure [2,27,28]. Since the discovery of B. burgdorferi as the Lyme disease agent, OspA has been a subject of intensive investigation [29]. In contrast, less is known about the role of OspB in the life cycle of *B. burgdorferi.* Previous studies have identified an *ospB* escape mutant in a clonal population of infectious B. burgdorferi with a single base change in the consensus ribosomal binding sequence and a single nucleotide deletion in the open reading frame of *ospB* gene [30]. These changes in the *ospB* gene resulted in reduced expression and truncation of this protein that diminished the penetration capability and infectivity of the spirochete in the human umbilical vein endothelium cells [30]. Several reports have indicated that OspB is present on the surface of B. burgdorferi within unfed ticks [31–33] and that OspB antibody inhibits B. burgdorferi colonization in I. scapularis gut [34]. Targeted gene disruption of *ospAB* operon inhibited B. burgdorferi colonization and persistence in the tick gut [35]. While this study highlighted an important role of OspA in spirochete-tick interaction in vivo, the independent role of OspB in the life cycle of spirochetes remains unclear.

Progress in the methodologies for genetic manipulation of virulent B. burgdorferi allows researchers to study the importance of specific B. burgdorferi genes that are required throughout the spirochete life cycle [36]. In the current study, we have analyzed the functional role of OspB during spirochete-tick interactions by generating an OspB-deficient *B. burgdorferi,* genetically complementing the mutant, and performing in vivo studies in ticks and mice.

## Results

### Generation of OspB-Deficient B. burgdorferi

**Figure 1:**
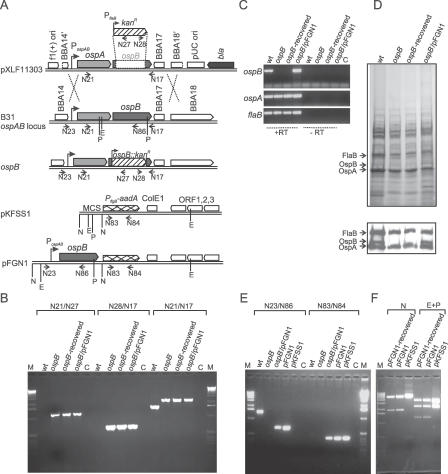
Characterization of the *ospB* Mutant and the OspB Complemented Mutant (A) Strategy for the inactivation of the *ospB* gene and for the generation of the OspB complemented mutant is shown. The top diagram represents the suicide vector pXLF11303 used for transformation into B. burgdorferi B31. Due to homologous recombination (double crossover) between the sequences of pXLF11303 and the native *ospAB* locus in B. burgdorferi B31, the Δ*ospB::kan* fragment is inserted into the genome, resulting in the generation of the *ospB* mutant (*ospB* ^−^). For the generation of the OspB complemented mutant, the P*_ospAB_* and *ospB* gene were PCR-amplified from B. burgdorferi B31 total genomic DNA and cloned into the multiple cloning site (MCS) of the shuttle vector pKFSS1 [40], resulting in the construct pFGN1 (bottom diagram). The relevant structures of the plasmid and genome are shown. Arrows represent positions of the oligonucleotides used for the PCR analysis. Primer names are retained as shown in Table 1. Vertical lines represent the restriction enzyme sites. E, EcoRI; N, NotI; and P, PstI. (B) PCR analyses of genomic DNA isolated from spirochetes were performed to confirm the inactivated *ospB* locus. wt, the wild-type isolate; *ospB,* the *ospB* mutant; *ospB* ^−^ , recovered-spirochetes recovered from mice infected with the *ospB* mutant; *ospB* ^−^/pFGN1, the OspB complemented mutant; M, 1-kb DNA ladder; C in (B and E) refers to PCR reactions without template DNA. (C) RT-PCR analysis of *ospA, ospB,* and *flaB* transcripts in spirochetes grown in BSK-H media. Shown is a gel image of RT-PCR reactions performed with (+RT) and without (−RT) reverse transcriptase. C refers to RT-PCR reactions without cDNA. (D) SDS-PAGE (top) and immunoblot (bottom) analysis of whole-cell lysates of spirochetes. Arrows indicate the expression of OspA, OspB, and FlaB. Immunoblotting analysis performed with monoclonal antibodies directed against OspB (mAb B22J), OspA (C3.78), and FlaB (H9729) were reported previously [22,50]. (E) PCR analyses of genomic DNA to confirm the OspB complementation. (F) Restriction digestion of pFGN1 plasmid recovered from the OspB complemented mutant (pFGN1-recovered) and plasmid controls pFGN1 and pKFSS1. Whole-cell lysate from the OspB complemented mutant was transformed into E. coli DH5α-competent cells and transformed clones were selected in the presence of spectinomycin (50 μg/ml). Plasmids isolated from E. coli cells were digested with NotI or EcoRI and PstI. Expected sizes of restrictive fragments are (i) 5,638 bp and 1,906 bp for NotI digestion of pFGN1; (ii) 5,638 bp and 453 bp for NotI digestion of pKFSS1; (iii) 3,840 bp, 2,413 bp, and 1,291 bp for EcoRI*-*PstI digestion of pFGN1; and (iv) 3,635 bp, 2,413 bp, and 43 bp for EcoRI*-*PstI digestion of pKFSS1.

To understand the function of B. burgdorferi OspB, an isogenic OspB-deficient mutant was generated from infectious B. burgdorferi B31 clone 5A11 by replacing a 554-base pair (bp) internal fragment of the *ospB* gene with a *Borrelia*-adapted kanamycin resistance cassette, kanAn, through homologous recombination (Figure 1). The construct used to inactivate the *ospB* gene, pXLF11303, is schematically shown in Figure 1A. After electroporation of clone 5A11 with pXLF11303, three kanamycin-resistant B. burgdorferi transformants were obtained, of which two were confirmed to be OspB-deficient by immunoblot (unpublished data). The plasmid content of these two mutants was analyzed and compared to that of the wild-type parental clone using an array-based assay [37], which showed that one of the mutants lost cp9 and the other lost lp28–1. Because the loss of cp9 has been shown to have little effect on B. burgdorferi infectivity in mice [38], we chose the *ospB* mutant lacking cp9 for further studies.

**Table 1:**
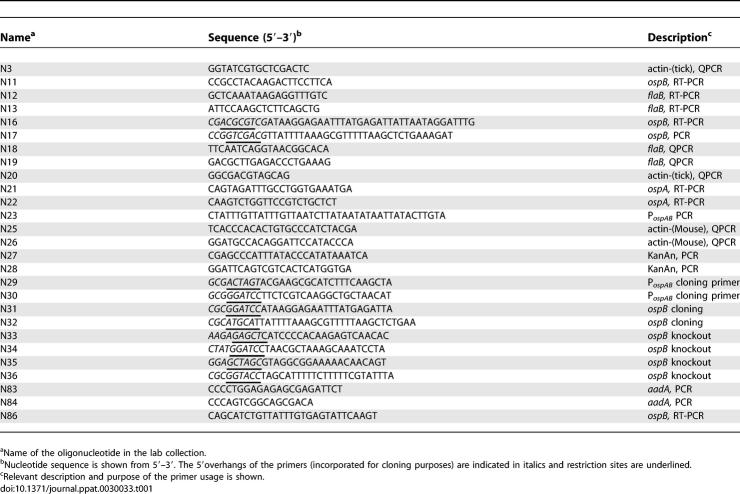
Oligonucleotides Used in This Study

To verify the desired genomic arrangements in the *ospB* mutant, a series of PCRs were performed (Figure 1B). The two PCRs using primer combinations N21/N27 and N28/N17 verified the left and the right junctions of the replacement in the *ospB* mutant. PCR with primer combination N21/N17 generated different sized DNA fragments from the wild-type strain and the *ospB* mutant, which is consistent with the replacement of the 554-bp internal fragment of the *ospB* gene with the 1,248-bp kanAn cassette. Collectively, these PCR results show that a double-crossover event had occurred in the mutant, resulting in the inactivation of the *ospB* gene. RT-PCR and immunoblot further showed that the *ospB* mutant lacked *ospB* mRNA and was OspB-deficient (Figure 1C and 1D). The *ospB* mutant had a similar total protein profile as well as comparable OspA mRNA and protein levels to that of the wild-type isolate (Figure 1C and 1D).

### The OspB-Deficient B. burgdorferi Is Infectious and Pathogenic in Mice

**Figure 2:**
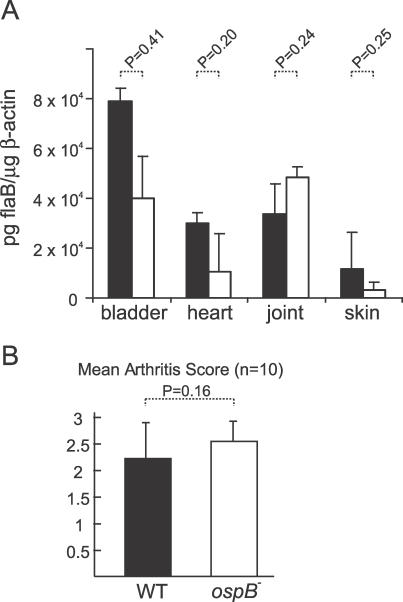
Influence of OspB Deficiency on B. burgdorferi Infectivity for Mice Spirochete burden (A) and joint swelling and inflammation (B) in C3H/HeN mice were quantified at 25 d after mice were needle-inoculated each with 10^5^ wild-type (black bars) or *ospB* mutant (white bars) spirochetes. Spirochete burden in mice tissues (bladder, heart, joint, and skin) was quantified by Q-PCR and shown as pg of *flaB* DNA per μg of mouse β-actin. Both tibiotarsal and knee joints of each mouse were scored on a scale of 0–3 for joint swelling, exudation of fibrin and leukocytes into joint spaces, synovial thickening due to proliferation, and hypertrophy of synovial cells: absent (0), mild (1), moderate (2), or severe (3). Statistical analysis from Student *t* test is also shown. Error bars define standard deviation (+) from the average value (mean).

To evaluate whether the loss of OspB expression affected the pathogenicity of the spirochete, we examined both the wild-type and the *ospB* mutant spirochetes in the murine model of Lyme borreliosis [39]. Groups of three C3H/HeN mice were challenged intradermally with in vitro-grown spirochetes, either the mutant or the wild-type *B. burgdorferi,* at a dose of 10^5^ spirochetes/mouse (see Materials and Methods for details). At 25 d post-inoculation, mice were sacrificed and spirochete infection was assessed by serology and in vitro culturing of the bladder and the spleen. The results of three independent experiments using a total of 18 mice, nine infected with wild-type strain and the other nine infected with the mutant strain, indicated that all mice seroconverted and were culture-positive for spirochetes (unpublished data). The *ospB* mutant spirochetes recovered from murine tissues remained OspB-deficient, indicating that no reversion had occurred (Figure 1B, 1C, and 1D). Mice infected with the wild-type or the *ospB* mutant B. burgdorferi had similar spirochete burdens in various tissues, including the bladder, heart, joints, and skin (*p* > 0.05, Figure 2A). Joint swelling and inflammation were similar in both groups of mice (*p* > 0.05, Figure 2B). Taken together, these data indicate that at the dose of 10^5^ spirochetes/mouse the *ospB* mutant is fully infectious and pathogenic in mice.

### The OspB-Deficient B. burgdorferi Enters but Cannot Colonize or Survive in I. scapularis

**Figure 3:**
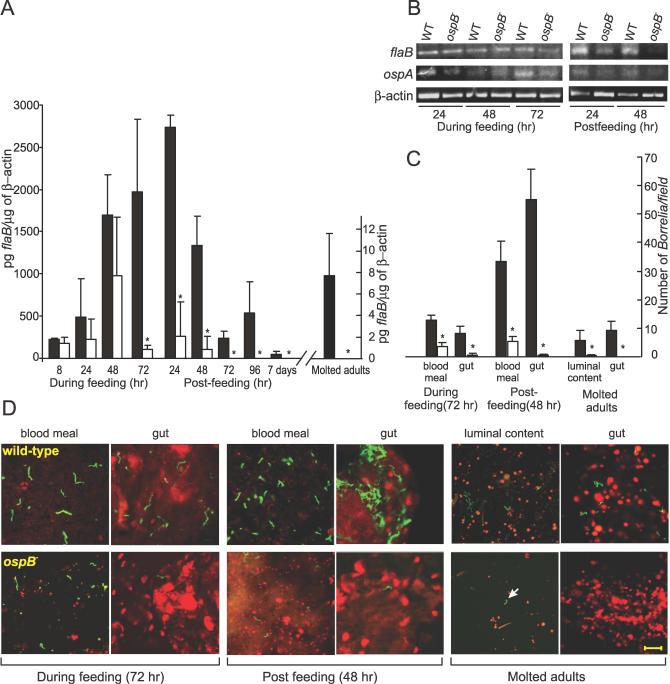
Influence of OspB-Deficiency on the Ability of B. burgdorferi to Survive and Colonize in Ticks (A) Results from the experiments with the ticks inoculated by feeding on infected mice are shown. The spirochete burden in nymphal and subsequently molted adult ticks is quantified by Q-RT-PCR. Value on Y-axis corresponds to pg of *flaB*/μg of tick *β-*actin in each cDNA sample. * indicates significant reduction (*p* < 0.05, Student *t* test) of the *ospB* mutant in ticks in comparison to the wild-type. (B) RT-PCR analysis of the RNA samples extracted from nymphs is shown. RT-PCR of *flaB* and *ospA* transcripts were used to determine the presence of spirochetes; tick *β-actin* was used as an internal control for RT-PCR. (C) Shown is the quantitative assessment of the number of spirochetes from 15 random microscopic field observations. Value on Y-axis corresponds to the number of spirochetes/microscopic field. Black and white bars in (A and C) indicate values for the wild-type and the *ospB* mutant spirochetes, respectively. Error bars define standard deviation (+) from the average value (mean). (D) Shown are the representative confocal images of the I. scapularis nymphal ticks (fed on mice infected with the wild-type or the *ospB* mutant B. burgdorferi at the indicated time points) and molted adult ticks. Tick blood meal (in nymphs) and luminal content (in adult ticks) and gut tissues were stained for B. burgdorferi with FITC-labeled anti-*Borrelia* antibody (green) and for tick cell nucleic acid with propidium iodide (red). Arrow shown in the luminal content sample of the molted adult tick indicates the only *ospB* mutant spirochete seen in 15 random microscopic fields. Scale 20 μm.

To determine whether the lack of OspB expression influenced the arthropod phase of the B. burgdorferi life cycle, uninfected nymphs were allowed to engorge on mice infected with either the wild-type or the OspB-deficient *B. burgdorferi.* Infection in mice was confirmed by positive *flaB* PCR from an ear punch biopsy. Three independent experiments were carried out with a total of 18 mice (nine infected with wild-type and the other nine infected with the mutant strain) and a total of 900 naïve nymphs (50 nymphs/mice). Five nymphs were removed from the murine skin at various time points during feeding, in order to examine the kinetics of spirochete migration, and the remaining nymphs were allowed to feed to repletion. Nymphs were subjected to Q-RT-PCR analyses to determine the spirochete burden (see Materials and Methods for details). At 8, 24, and 48 h during feeding, the viable spirochete burden in the ticks fed on the wild-type or the *ospB* mutant B. burgdorferi-infected mice were comparable (*p* > 0.05, Figure 3A). From 72 h (during feeding) onward, the *ospB* mutant spirochete levels were dramatically reduced in ticks compared with controls (*p* < 0.03, Figure 3A). RT-PCR for *flaB* and *ospA* transcripts yielded similar results (Figure 3B).

The observed reduction of spirochetes in ticks fed on mice infected with the OspB-deficient B. burgdorferi was further evaluated by immunofluorescence analysis (IFA) of a subset of the nymphs that were collected at various time points. Tick gut luminal contents (including the blood meal) as well as gut tissues washed free of luminal contents were prepared and subjected to IFA (see Materials and Methods for details). At 72 h of tick feeding, a striking number of wild-type spirochetes were detected in both blood meal (∼13 spirochetes/microscopic field) and gut (∼8 spirochetes/microscopic field) tissue samples (Figure 3C and 3D). In contrast, the spirochete number was drastically reduced (∼4-fold in blood meal and ∼16-fold in gut tissue) in nymphs fed on the *ospB* mutant-infected mice (See 72-h panel in Figure 3C and 3D). Of note, the number of OspB-deficient spirochetes adhering to the tick gut was significantly less (∼7-fold, *p* < 0.0001) in comparison to the number of the OspB-deficient spirochetes in the blood meal sample (Figure 3D). IFA of ticks from the 48-h post-feeding phase further corroborated these findings (Figure 3C and 3D).

Furthermore, a subset of fully engorged nymphs was allowed to molt to the adult stage to determine whether the diminished capacity of *ospB* mutant to colonize the tick gut was also reflected in the adult stage. The wild-type spirochetes were readily detected in both luminal content and gut sample (Figure 3C and 3D). In contrast, *ospB* mutant spirochetes were not detected in the gut sample and out of 15 microscopic field observations only one *ospB* mutant spirochete was seen in the luminal content sample (*p* < 0.0001, Figure 3C and 3D). A quantitative RT-PCR analysis also corroborated the results (*p* < 0.02, Figure 3A). Thus, these observations collectively show that the *ospB* mutant spirochetes and the wild-type B. burgdorferi are acquired by ticks at the same rate (note results at nymphal stage 8, 24, and 48 h during feeding), but the *ospB* mutant spirochetes are not able to persist within the luminal content or fully adhere to the tick gut.

### Complementation of the *ospB* Mutant

To further address whether the loss of OspB expression results in a defect of B. burgdorferi colonization and survival in the ticks, we constructed a strain of the mutant complemented in trans with a wild-type copy of the *ospB* gene. The promoter of the *ospAB* operon was fused to the *ospB* gene and cloned into the shuttle vector pKFSS1 [40], resulting in the construct designated as pFGN1 (Figure 1A). Electroporation of the *ospB* mutant with pFGN1 resulted in three positive clones that grew in the BSK-H media supplemented with streptomycin and kanamycin, of which one clone had lost both lp25 and lp28–1 plasmids and the other two clones showed identical endogenous plasmid profiles as its parental isolate (unpublished data). Furthermore, to determine whether these two clones harbored pFGN1 and were indeed from the *ospB* mutant, total DNA of these complemented clones was examined by PCR amplification. The three PCRs using primer combinations N21/N27, N28/N17, and N21/N17 confirmed the inactivated *ospB* locus (representative result shown in Figure 1B). PCR with primer combination N23/N86 generated different sized DNA fragments from the wild-type and the complemented strains, which confirmed the presence of P*_ospAB_*-*ospB* gene fusion, and PCR amplification of the internal sequences of the *aadA* gene with primer combinations N83/N84 further confirmed the presence of pFGN1 in the transcomplemented strains (representative result shown in 1E). Collectively, these PCR results revealed the expected amplicons with both complemented clones in all the PCR reactions in comparison to the wild-type and the *ospB* mutant. We chose one of these two clones, designated as the OspB complemented strain (*ospB* ^−^/pFGN1) for further analysis. OspB expression was detected at both the mRNA and the protein levels in the OspB complemented strain (Figure 1C and 1D). Furthermore, to confirm that the OspB complemented strain contained the pFGN1 plasmid, whole-cell lysates of kanamycin- and streptomycin-resistant cells were used to transform Escherichia coli DH5α-competent cells. Plasmid was then rescued from these E. coli transformants, and restrictive digestions were performed to verify the recovery of pFGN1 (Figure 1F). Immunoblot with mAb B22J was also performed to confirm the presence of OspB protein in these transformed E. coli cells (unpublished data).

**Figure 4:**
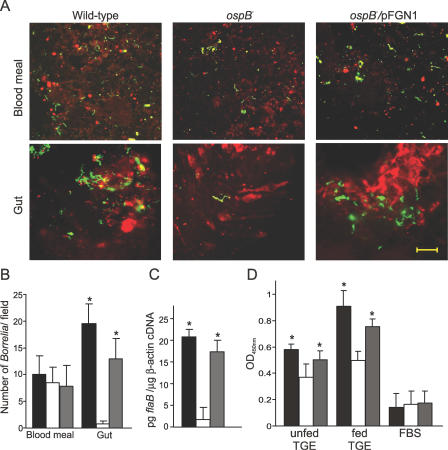
OspB Complementation Restores B. burgdorferi Ability to Survive and Colonize Ticks Results from the experiments with the ticks inoculated by microinjection via rectal aperture are shown. Approximately 10^3^ spirochetes were microinjected into each tick. (A) Shown are representative confocal microscopic images of the blood meal and gut samples of microinjected nymphs at 48 h during feeding. Samples were stained with FITC-labeled anti-*Borrelia* antibody (green), and tick cell nucleic acid was stained with propidium iodide (red). Scale 20 μM. (B) Average value of number of spirochetes from 15 random microscopic field observations is shown. (C) Q-RT-PCR for the total RNA extracted from microinjected nymphs collected at 48 h during feeding is shown. Values on Y-axis represent pg *flaB*/μg tick *β-*actin cDNA. (D) Shown are the readings from an in vitro binding assay of the wild-type, the *ospB* mutant, and the OspB complemented mutant B. burgdorferi to TGE- and FBS-coated wells (see Materials and Methods for details). Values on Y-axis are sample absorbance measured at 450 nm of wavelength. The values shown are the averages of four independent experiments. Black, open, and gray bars in (B, C, and D) indicate values for the wild-type, the *ospB* mutant, and the OspB complemented mutant spirochetes, respectively. Error bars define standard deviation (+) from the average value (mean). * indicates values that are statistically significant (*p* < 0.05, Student *t* test) in comparison to the *ospB* mutant.

Experimentally infected nymphs were prepared by microinjection of cultured spirochetes into the rectal aperture of uninfected nymphs as previously described [35]. Three independent experiments were carried out with a total of nine C3H/HeN naïve mice (three mice for feeding nymphs microinjected with wild-type, three for *ospB* mutant, and three for OspB complemented strain) and a total of 135 nymphs (15 nymphs/mice). Eight nymphs were forcibly removed during feeding (48 h) and analyzed by IFA and Q-RT-PCR. The results of the confocal microscopy revealed that, in contrast to the *ospB* mutant, the OspB complemented strain readily colonized the tick gut tissue (*p* < 0.0001), albeit to a lower level than the wild-type isolate (Figure 4A and 4B). No significant difference was seen in the blood meal samples of the nymphs infected with the wild-type, the *ospB* mutant, or the OspB complemented strain (Figure 4A and 4B). Quantitative RT-PCR analysis of cDNA samples also supported the microscopic observations that showed a significant higher level in the persistence of the OspB complemented strain in comparison to the *ospB* mutant (*p* < 0.001, Figure 4C).

### The OspB-Deficient B. burgdorferi Show Reduced Binding In Vitro to I. scapularis Gut

To further support the role of OspB in the attachment of B. burgdorferi to tick gut tissue, we performed an in vitro binding assay to the tick gut extract (TGE) prepared separately from flat-nymphal ticks and fed-nymphal ticks with the wild-type, the *ospB* mutant, and the OspB complemented strains (See Materials and Methods for details). The results revealed that the binding of the *ospB* mutant to TGE from flat nymphs and fed nymphs is significantly reduced by ∼40% and 60%, respectively, in comparison to the wild-type B. burgdorferi (*p* < 0.0001, Figure 4D). In contrast, the OspB complemented strain showed a significant increase in the binding to both TGE in comparison to the *ospB* mutant (*p* < 0.0001) and was comparable to the wild-type spirochetes (Figure 4D). Taken together, these data from Figure 4 show that genetic complementation of the *ospB* mutant with a wild-type copy of the *ospB* gene restores the defects seen in the colonization and survival of the *ospB* mutant inside the ticks and can restore B. burgdorferi binding to the TGE.

## Discussion

B. burgdorferi present an amazing variety of Osps that enable them to invade, colonize, and persist in environmental niches such as those inside vertebrates or ticks [23,26,41]. OspA, OspB, OspC, and DbpA are several of the major lipoproteins of B. burgdorferi that are differentially expressed in response to the varying environmental conditions [23,26,41]. B. burgdorferi upregulates OspA and OspB upon entry into ticks, and OspA contributes to the colonization of spirochetes within the vector gut [22]. Since *ospA* and *ospB* are cotranscribed [25,26] and colocalized on the bacterial surface [42], we speculated that OspB might also function for B. burgdorferi within ticks. To determine the precise role of OspB in the life cycle of *B. burgdorferi,* we have generated an OspB-deficient isogenic isolate of *B. burgdorferi.* Our data show that OspB facilitates the colonization and survival of B. burgdorferi within ticks.

While significant research has focused on the biological role of OspA in spirochete life cycle, relatively little information is available on the role of OspB in the life cycle of *B. burgdorferi.* Our in vivo studies with the OspB-deficient B. burgdorferi show that OspB is essential for the colonization and persistence of B. burgdorferi in ticks. During tick feeding, the *ospB* mutant and the wild-type B. burgdorferi enter the ticks from infected mice at the same rate. However, after feeding, the *ospB* mutant spirochetes are unable to persist within the blood meal or fully adhere to the tick gut, which also leads to a significant reduction in the number of spirochetes in the molted adult ticks (Figure 3). The binding of residual OspB-deficient spirochetes to the tick gut could be attributed to OspA, as the level of the OspA is unaltered in the OspB-deficient spirochetes (Figure 1). The luminal face of the gut epithelium is covered by a dense array of glycoproteins that may act as “receptor-buffet” for many pathogens [43]. Some of these glycoproteins are involved in general tissue structure and digestion [43,44] and some are involved in innate immunity [45,46]. Our surprising finding that the reduced ability of the *ospB* mutant to attach to the gut epithelium and its subsequent clearance in the gut may suggest that adherence to tick gut cells also is critical for some as yet unknown aspect(s) of spirochete viability.

Our in vivo analysis showed that in contrast to the *ospB* mutant, the OspB complemented strain readily colonized tick gut tissue and showed a drastic increase in its persistence within ticks, which was comparable to the wild-type isolate (Figure 4). Furthermore, our in vitro binding assays with the TGE also supported the in vivo analysis, indicating that in contrast to the *ospB* mutant, the transcomplemented strain binds with a greater affinity to the TGE. In addition, the difference between the wild-type and the *ospB* mutant spirochetes in binding to the fed TGE is significantly higher in comparison to the unfed TGE, suggesting that the levels of expression of putative OspB gut receptor proteins/glycolipids might increase during feeding. Furthermore, our in vitro binding data correlated with a previous study [34] showing that the B. burgdorferi N40 OspB protein binds significantly to the TGE. Overall, our studies solidify a great body of experimentation implicating an important role of OspB in the attachment of B. burgdorferi to the tick gut.

Yang and co-workers recently examined the role of the *ospAB* locus in the infectious life cycle of B. burgdorferi [35]. This was accomplished by the generation of an *ospAB* double mutant from B. burgdorferi strain BbAH130 (infectious clone recovered after plating B. burgdorferi strain 297), and it was found that disruption of both the *ospA* and *ospB* genes had no observable effect on the ability of spirochetes to establish infection in mice, whereas the locus is critically essential for colonization of the tick gut [35]. Spirochetes deficient for both OspA and OspB entered ticks but were unable to persist within ticks for a long time [35]. Furthermore, complementation of the *ospAB* double mutant with both OspA and OspB expression restores the ability of B. burgdorferi to colonize the gut [35]. On the other hand, complementation with the *ospA* gene alone could only partially restore (50%–60% in comparison to the wild-type) the colonization defect of the *ospAB* mutant [35], suggesting that OspB expression is also required for the complete restoration of the defect. A comparison of our data with the prior study [35] indicated that in contrast to the *ospAB* double mutant complemented with the *ospA* gene alone, the *ospB* mutant analyzed in our study was significantly impaired in its persistence in the tick gut. These variations could have been the results of (i) the different B. burgdorferi strains used in the studies (B31 5A11 versus 297 BbAH130) and (ii) the relative OspA expression in the *ospAB* double mutant complemented with the *ospA* gene (on a circular plasmid) compared to the *ospB* mutant analyzed in our study. Overall, our studies in conjunction with the previous studies [35] show that (i) absence of OspB alone could result in severe impairment in colonization and persistence, and (ii) absence of both OspA and OspB could lead to the complete impairment in colonization and persistence of B. burgdorferi in ticks. In an evolutionary perspective, the conservation of *ospB* in the genome of B. burgdorferi is the result of positive selection pressure [23,24], and thus OspB must be of intrinsic value to the organism. Our studies suggest that the function of OspB and OspA are codependent.

OspA and OspB share approximately 50% identity and 62% similarity in their amino acid sequences [2]. The crystal structures of OspA and the C-terminal region of OspB have been determined [27,28]. Comparison of the crystal structure of OspA and C-terminal region of OspB shows that these two molecules are quite similar [27,28]. The C-terminal region of OspB adopts the same fold as is observed for the C-terminal half of OspA [28]. Li and co-workers have identified that the C-terminal barrel domain in OspA is a trio of partially buried charged residues: Arg 139 from beta-strand 10, Glu-160 from beta-strand 12, and Lys-189 from beta-strand 15 [27]. The barrel domain of the OspA/B fold features a prominent cavity, in which the first two residues are strictly conserved in both OspA and OspB; position 189 is nearly always Lys in OspA and Arg in OspB [27,28]. Studies from Li et al. (1997) and Becker et al. (2005) have proposed that the cavity in the OspA/B barrel domain might be a ligand binding site for a small peptide, linear saccharide, or an exposed protein loop [27,28]. Furthermore, the mapping of amino acid sequences required for OspA binding to the tick gut showed that the residues 85–103 and 229–247 are important [47]. The percent similarity (identity) for the two amino acid stretches are 63 (68) and 84 (79) in OspB amino acid sequence at positions 110–128 and 252–270, respectively [34,47]. Given the structural and amino acid sequence conservation of OspA and OspB, it is possible that both lipoproteins recognize either the same target or closely related targets. It has recently been shown that OspB antibodies prevent B. burgdorferi colonization of I. scapularis gut [34]. Because of the high structural similarities between OspA and OspB, it could be reasoned that the OspB antibodies may bind to several epitopes of OspB on the B. burgdorferi surface, and steric hindrance might then interfere with OspB binding to the tick gut; or it is possible that steric hindrance by OspB antibodies also affected OspA-mediated binding of spirochetes to the tick gut [34]. Thus, our studies in conjunction with the previous reported study [34] raises interesting questions regarding the potential of antibody binding interfering with spirochete adherence in ticks. In the in vitro-grown B. burgdorferi cultures, the expression of OspB is lower than OspA (Figure 1D). Since OspA is also involved in the attachment of spirochetes to the tick gut and since we have found that OspB-deficient spirochetes are unable to attach to the tick gut, it is possible that disruption of the OspB resulted in the interference of the OspA-mediated attachment to TROSPA. Three scenarios may be envisioned that may elucidate the possible involvement of OspB in the OspA-TROSPA interactions. Firstly, OspB may directly associate with either OspA or TROSPA and may form a complex structure that is required for the tight attachment of B. burgdorferi to the tick gut. Secondly, OspB may bind to its own receptor within the gut and this interaction might be required for TROSPA to interact with OspA. Finally, OspA and OspB might bind to separate TROSPA molecules on the gut epithelium and both these interactions might be required for the tight attachment of B. burgdorferi to the tick gut. With any of these three models, our finding that OspB-deficient spirochetes were unable to colonize or persist in tick gut is significant because it suggests a possible synergistic interaction between OspA, OspB, and TROSPA.

In summary, these data suggest that OspB plays a critical role for B. burgdorferi adherence and persistence in ticks. These studies are not only important in understanding significant roles of spirochete ligands (such as OspB) in spirochete colonization and survival at arthropod-pathogen interface, but they also enhance our knowledge in the development of new therapeutic strategies, such as new transmission blocking vaccines that may be useful to combat B. burgdorferi infection.

## Materials and Methods

### Bacterial isolates and ticks.

B. burgdorferi isolate B31 infectious clone 5A11 that lacks lp5 and contains all other 20 plasmids [38,48] was used throughout, and will be referred to herein as the wild-type B. burgdorferi. This B. burgdorferi isolate, its *ospB* deletion mutant, and the OspB complemented mutant (*ospB* ^−^/pFGN1) were cultivated in vitro at 33 °C in Barbour-Stoenner-Kelly complete media (BSK-H, Sigma, http://www.sigmaaldrich.com). Where necessary, antibiotics kanamycin and streptomycin were added to a final concentration of 350 μg/ml and 50 μg/ml, respectively. Spirochetes from in vitro cultures were needle-inoculated (intradermally) at a dose of 10^5^ spirochetes/mouse and were recovered from cultures of ear punch biopsies at 2 wk after inoculation. E. coli strain DH5α (Invitrogen, http://www.invitrogen.com) was used as a cloning host. Unfed I. scapularis nymphs were obtained from a colony of I. scapularis ticks maintained by Dr. Durland Fish and Dr. Fred S. Kantor at Yale University.

### Construction of mutagenic plasmid and OspB-deficient *B. burgdorferi.*

The *Borrelia*-adapted kanamycin resistance cassette, kanAn, driven by the *flaB* promoter, was excised from pTAkanAn [49] by EcoRI digestion and cloned into the *Eco*RI site of pBluescript (Stratagene, http://www.stratagene.com). The resulting construct, designated pXLF10601, contains two multi-cloning sites flanking the kanAn cassette, which allows efficient cloning of the 5′ and the 3′ arms required for homologous recombination. PCR primers N33/N34 and N35/N36 (Table 1) for the construction of mutant spirochetes were designed to amplify the 5′ arm (bp 8883–10257 of lp54) and the 3′ arm (bp 10812–12015 of lp54), respectively. The oligonucleotides were designed based on the sequenced genome of B. burgdorferi B31 isolate M1 [2]. The restriction sites designed in the primers allow directional cloning of the 5′ arm into the SacI-BamHI site and the 3′ arm into the NheI-KpnI site of pXLF10601 to generate the construct pXLF11303 (Figure 1A).

To generate an *ospB* mutant, 20 μg of pXLF11303 was electroporated into B. burgdorferi B31 strain and the mixture was incubated at 33 °C overnight in 20 ml of BSK-H medium. After an 18–24 h recovery period, 20 ml of BSK-H medium containing 700 μg/ml kanamycin was added (final concentration of kanamycin, 350 μg/ml) and 200 μl aliquots were dispensed into two 96-well plates. After 2–3 wk of incubation at 33 °C in a CO_2_ incubator, the wells that contained kanamycin-resistant (Kan^R^) clones were identified by the color change of the culture medium. The presence of viable spirochetes in these wells was subsequently verified by dark-field microscopy. The homologous recombination between the sequences of pXLF11303 and of the native *ospAB* operon results in the excision of the *ospB* gene with the integration of the Δ*ospB*::Kan fragment (Figure 1A). The *ospB* mutant was confirmed by PCR with primers N21/N27, N28/N17, and N21/N17 (Table 1 and Figure 1B); RT-PCR (with N16/N11 primers); and Western blot analysis with monoclonal antibody B22J [50] (Figure 1C and 1D). The plasmid profile of the *ospB* mutants was examined by PCR and array hybridization as previously described [37,38].

### Complementation of the *ospB* mutant.

DNA fragments carrying the wild-type copy of promoter sequences of *ospAB* operon (P*_ospAB_*) and the *ospB* gene were PCR-amplified from the B. burgdorferi genomic DNA using primers N29/N30 and N31/N32, respectively (Table 1). The PCR products were subsequently cloned into the vector pKFSS1 [40] at the XbaI-PstI site, resulting in pFGN1 (Figure 1A). 20 μg of pFGN1 DNA was electroporated into competent cells of the *ospB* mutant and the mixture was incubated at 33 °C overnight in 20 ml of BSK-H medium. After an 18–24 h recovery period, 20 ml of BSK-H medium containing 100 μg/ml streptomycin and 700 μg/ml kanamycin was added (final concentration of streptomycin and kanamycin was 50 μg/ml and 350 μg/ml, respectively) and 200 μl-aliquots were distributed into two 96-well plates. After 2–3 wk of incubation, streptomycin- and kanamycin-resistant clones were selected and analyzed by PCR with primers N23/86 and N83/N84 to detect the P*_ospAB_*-*ospB* and the *aadA* fragments, respectively. RT-PCR with N16/N11 primers and immunoblot with mAb B22J were also performed to confirm the presence of *ospB* transcripts and OspB protein, respectively (Figure 1C and 1D). Plasmid content of the complemented clones was analyzed by PCR as previously described [38,51].

### B. burgdorferi infection of mice via needle inoculation.

10^5^ spirochetes from an in vitro-grown culture were needle-inoculated into the groups of three 3-wk-old C3H/HeN mice. 2 wk later, ear punch biopsies were taken and DNA was extracted for PCR with primers N18 and N19 (*flaB* gene primers, Table 1) to confirm the infectivity. After 3 wk of infection, tibiotarsal and knee joints were evaluated for swelling [39]. Mice were sacrificed after the tick feeding and evaluation of joint swelling, and tissues such as skin, heart, bladder, and joints were harvested for DNA extraction. In parallel, for recovery of B. burgdorferi from mice, a part of bladder and spleen was inoculated into BSK-H medium and cultivated at 33 °C.

### B. burgdorferi transmission from infected mice to naïve nymphs.

After a 3-wk inoculation of B. burgdorferi into mice, nymphal I. scapularis ticks (50 ticks/mice) were allowed to feed on B. burgdorferi-infected mice. Partially fed nymphs (five nymphs/time point) were removed from the skin of mice at various time points during feeding (8 h, 24 h, 48 h, and 72 h). Remaining nymphs were allowed to feed to repletion and were collected at 24 h, 48 h, and 72 h following up to 7 d post-feeding (corresponding to 96 h, 120 h, 144 h, and 10 d, respectively, from the feeding start time point). Three to five guts from each group of nymphs were microscopically dissected in 20 μl phosphate-buffered saline (PBS) and washed to remove luminal contents and unbound bacteria. In addition, luminal contents (including the blood) from the gut were separately isolated from the fed ticks and subjected to immunofluorescence confocal microscopy as described [35,52]. The spirochete burden in ticks was further analyzed by quantitative RT-PCR.

### Molting of infected nymphs into adults.

Nymphal ticks were allowed to feed to repletion on B. burgdorferi-infected mice. Engorged nymphs were then collected and transferred into 10-ml glass vials with vented lid and stored at 22 °C incubator with a relative humidity of 97% and 16 h:8 h light:dark photoperiod. Engorged nymphs were allowed to molt to the adult stage for 6–8 wk. Five to seven guts from adults were microscopically dissected, and guts and luminal contents were subjected to immunofluorescence confocal microscopy as described [35,52]. Spirochete burden in adult ticks was further evaluated by quantitative RT-PCR analysis.

### Confocal microscopy and immunofluorescence.

The washed gut and luminal contents were subjected to immunofluorescence confocal microscopy as described [35,52]. Briefly, gut and luminal content samples were blocked with PBS containing 0.05% Tween 20 and 5% goat serum for 1 h at 37 °C and then incubated for 1 h at 37 °C with FITC-labeled anti-*Borrelia* antibody (KPL, http://www.kpl.com). Samples were subsequently stained with propidium iodide (20 μg/ml) for 3 min at 37 °C followed by mounting with SlowFade-Antifade kit (Invitrogen). The tissues were viewed using a Zeiss LSM 510 scanning laser confocal microscope equipped with an argon/krypton laser (Zeiss, http://www.zeiss.com).

### Microinjection of B. burgdorferi into ticks.

Microinjection of B. burgdorferi into the ticks was performed as described [35]. Briefly, B. burgdorferi isolates (wild-type, *ospB* mutant, and OspB complemented mutant) were cultivated under normal conditions in BSK-H medium with or without antibiotics. Log-phase cultures were centrifuged and concentrated in a fresh BSK-H media to a density of 10^9^ spirochetes per ml. 1 μl of the re-suspended cultures was then loaded into a 1-mm diameter glass capillary needle (World Precision Instruments, http://www.wpiinc.com) and approximately 1 nl (10^3^ spirochetes) was injected into each tick via the rectal aperture using femtojet microinjector system (Eppendorf AG, http://www.eppendorf.com) as described [22,35]. 15 microinjected ticks were reared in a humid chamber for 12 h and fed on naïve mice. Five to seven partially fed ticks were detached during feeding at 48 h. The spirochete burden in ticks was further analyzed by confocal microscopy and quantitative RT-PCR.

### PCR.

For RT-PCR analysis of B. burgdorferi grown in vitro, total RNA was isolated from B. burgdorferi B31 and its derived mutants using Nucleospin RNAII kit (BD Biosciences, Clontech, http://www.clontech.com) and converted to cDNA using iScript cDNA synthesis kit (Bio-Rad, http://www.bio-rad.com). RT-PCR for the *ospB, ospA,* and *flaB* genes was performed using the primer combinations N16/N11, N21/N22, and N12/N13, respectively (Table 1). For the RT-PCR analysis of B. burgdorferi RNA in ticks, RNA from the fed-tick extracts (three nymphs/group) was extracted and converted to cDNA. The cDNA was used as a template for the amplification of *flaB* using primers N18 and N19 and for tick *β-actin,* using primers N3 and N20.

For the quantification of the spirochete burden in mice and ticks, quantitative PCR (Q-PCR) and quantitative RT-PCR (Q-RT-PCR) analysis was performed, respectively, as described [22,35]. The 225-bp *flaB* amplicons were quantified using the oligonucleotides N18 and N19 and iQ-SYBR Green Supermix (Bio-Rad). The reaction conditions are initial 95 °C for 10 min followed by 94 °C for 30 s, 60 °C for 1 min, 72 °C for 1 min for 35 cycles. As an internal control and to normalize the amount of template, tick *actin* amplicons were quantified using N3/N20 (for tick samples), and mouse *β-actin* amplicons were quantified using the oligonucleotides N25 and N26 (for mice samples). Standard curves for *flaB,* tick *actin,* and mouse *β-actin* were prepared using 10-fold serial dilutions of known quantities (4 ng–0.4 fg for pCR2.1-*flaB;* 5 ng–0.5 fg for tick *β-actin;* 5 ng–0.5 fg for mouse *β-actin*).

### Spirochete binding to TGE.

Guts from flat-nymphal ticks (40 ticks) and fed-nymphal ticks (25 ticks) were dissected in PBS and homogenized on ice using Kontes micro homogenizer (VWR Scientific Products, http://www.vwrsp.com). Total protein concentrations in the TGE were determined using the Bio-Rad Protein Assay Kit (Bio-Rad Laboratories). 100 μl of TGE (5 μg/ml) in PBS was used to coat the wells of 96-well plates (Nunc, http://www.nuncbrand.com). Control wells were coated with fetal bovine serum (FBS) (10 μg/ml). The coated wells were incubated for 4 h at 33 °C with 10^7^ spirochetes per well in PBS-Tween 20 supplemented with 5% FBS. Unbound spirochetes were washed away with PBS-Tween, followed by incubation for 1 h at 33 °C with FITC-labeled anti-*Borrelia* antibody (KPL). Binding was detected using anti-FITC IgG-horseradish peroxidase (Amersham, http://www.gehealthcare.com) as a secondary reagent and TMB microwell peroxidase substrate (KPL) was used for color development. The reactions were stopped after 15 min incubation using TMB stop solution (KPL) and optical density (OD) was read at 450 nm.

### Antibodies and protein analysis.

For sodium dodecyl sulfate-polyacrylamide gel electrophoresis (SDS-PAGE) analysis, 10 ml of actively growing spirochetes (10^7^ cells/ml) were centrifuged and re-suspended in 25 μl Laemmli Sample buffer (Bio-Rad). Samples were incubated in boiling water for 5 min and loaded onto 12% SDS-PAGE gels. Gels were stained with Simply Blue SafeStain (Invitrogen) and processed according to the manufacturer's instruction. Immunoblotting analysis was performed with the protein extracts from 10^7^ spirochetes and processed as described [52]. Monoclonal antibodies directed against OspB (mAb B22J), OspA (C3.78), and FlaB (H9729) were reported previously [22,50].

## Supporting Information

### Accession Numbers

The GenBank (http://www.ncbi.nlm.nih.gov/Genbank) accession numbers for B. burgdorferi B31 isolate M1 are NC_001318 for the chromosomal genome sequence and NC_001857 for the lp54 sequence.

## Abbreviations

bp: base pair
IFA: immunofluorescence analysis
lp: linear plasmid
Osp: outer surface protein
s.l.: senso lato
TGE: tick gut extract
